# Dentures for V-Shaped Arches

**Published:** 1906-06

**Authors:** W. O. Garside

**Affiliations:** Montgomery.


### DENTURES FOR V-SHAPED ARCHES.

By W. C. Garside, D. D. S., Montgomery.

Alabama Dental Association.

The first thing you do is to make a nice little model and carve on each side of the median line a small suction chamber. By following this idea, you will be surprised at the way the plate will cling there.

I would like to give you a point in taking the bite. I would suggest using modeling compound; then you get a base plate that is reliable, and you can depend on it.

